# PRMDA: personalized recommendation-based MiRNA-disease association prediction

**DOI:** 10.18632/oncotarget.20996

**Published:** 2017-09-18

**Authors:** Zhu-Hong You, Luo-Pin Wang, Xing Chen, Shanwen Zhang, Xiao-Fang Li, Gui-Ying Yan, Zheng-Wei Li

**Affiliations:** ^1^ Department of Information Engineering, Xijing University, Xi’an, China; ^2^ International Software School, Wuhan University, Wuhan, China; ^3^ School of Information and Control Engineering, China University of Mining and Technology, Xuzhou, China; ^4^ Academy of Mathematics and Systems Science, Chinese Academy of Sciences, Beijing, China; ^5^ School of Computer Science and Technology, Hefei, China

**Keywords:** miRNA, disease, miRNA-disease association, personalized recommendation

## Abstract

Recently, researchers have been increasingly focusing on microRNAs (miRNAs) with accumulating evidence indicating that miRNAs serve as a vital role in various biological processes and dysfunctions of miRNAs are closely related with human complex diseases. Predicting potential associations between miRNAs and diseases is attached considerable significance in the domains of biology, medicine, and bioinformatics. In this study, we developed a computational model of Personalized Recommendation-based MiRNA-Disease Association prediction (PRMDA) to predict potential related miRNA for all diseases by implementing personalized recommendation-based algorithm based on integrated similarity for diseases and miRNAs. PRMDA is a global method capable of prioritizing candidate miRNAs for all diseases simultaneously. Moreover, the model could be applied to diseases without any known associated miRNAs. PRMDA obtained AUC of 0.8315 based on leave-one-out cross validation, which demonstrated that PRMDA could be regarded as a reliable tool for miRNA-disease association prediction. Besides, we implemented PRMDA on the HMDD V1.0 and HMDD V2.0 databases for three kinds of case studies about five important human cancers in order to test the performance of the model from different perspectives. As a result, 92%, 94%, 88%, 96% and 88% out of the top 50 candidate miRNAs predicted by PRMDA for Colon Neoplasms, Esophageal Neoplasms, Lymphoma, Lung Neoplasms and Breast Neoplasms, respectively, were confirmed by experimental reports.

## INTRODUCTION

Discovered in *Caenorhabditis elegans* at first, microRNAs (miRNAs) are a highly profuse class of short, with length of 21–24 nucleotides, endogenous single-stranded non-coding RNAs (ncRNAs) [1, 2]. Due to the diversity in sequence and expression patterns, miRNAs play important roles in regulating genes in both animals and vegetation by targeting miRNAs for cleavage or translational repression [3, 4]. The first two detected miRNAs lin-4 and let-7 are considered to be unique when first described, which were found to control developmental timing in Caenorhabditis elegans. However, several following findings suggested that miRNA genes were probably one of the most phylogenetically numerous and miscellaneous classes of ncRNA genes [5–7]. Since the discovery of the first two miRNAs, thousands of miRNAs have been discovered in the eukaryote ranging from fungus to mammals on the basis of copious experimental implementations and computational models in the past few years. With increasing genetic and bioinformatics analysis, researchers have found that by yielding a negative impact on the expression level of their target genes, miRNAs work through two different mechanisms: the miRNAs could perfectly or near-perfectly bind to their binding sequences within the 3′ untranslated regions (UTR) of their target mRNAs to indirectly induce cleavage of mRNA or control gene expression at the translational phase through imperfect target matching [8]. Furthermore, more and more evidences accumulated by substantial experiments indicate that miRNAs have a significant impact on various crucial cellular processes including propagation, differentiation, development, apoptosis, transduction, metabolism, viral infection and so on [4, 9–15]. Besides, plenty of studies have shown that miRNA mutations or mis-expression are closely related with various human cancers, indicating that miRNAs could perform as tumor suppressors and oncogenes [16]. For instance, Zhang *et al.* confirmed that downregulation of miRNA-181d, probably through reverse regulation on a microRNA-181d gene (Na+/K+ transporting ATPase interacting 2), could suppress the development of pancreatic cancer cell lines [17]. MiRNA is also common in hepatocellular carcinoma, an example is that Au SL *et al.*’s finding suggests that enhancer of zeste homolog 2 (EZH2) exerts its prometastatic function through epigenetic silencing of multiple tumor suppressor miRNAs including miR-101, miR-139-5p, let-7c, miR-125b, and miR-200b [18]. For lung cancer, one of the pivotal causes contributing to most cancer deaths in the United States, it has been verified that regular process of DNA methylation reinstatement, re-expression of methylation-silenced tumor suppressor genes, and inhibition of tumorigenicity was owing to the compulsory miR-29 expression in cell lines of lung cancer [19]. MiRNA expression was proved to be related to tumor formation, progression, development as well as reaction to treatment by amassing experimental evidence, from which we could deduce that miRNA has the potential practical application as biomarkers for diagnose, prognosis and prediction [20]. Lu *et al.* conducted a comprehensive analysis to the human miRNA-disease association database. As a result, the analysis unveiled significantly statistical patterns of miRNA-disease associations [21]. Taking a considerable number of biological databases related with miRNA into consideration, developing innovative and efficient computational models to identify possible miRNA-disease associations is urgently required. In Recent years, more and more new miRNAs and diseases have been discovered by researchers with the development of technology. Meanwhile, substantial number of associations between miRNAs and diseases remain to be identified. There is no doubt that prioritizing related-diseases and related-miRNAs for newly discovered miRNAs and diseases could effectively contribute to promoting disease biomarker detection for the prevention, diagnosis and treatment of human diseases [22]. It is also considered as a critical function for a method of identifying miRNA-disease associations.

Plenty of computational models for potential miRNA-disease association prediction have been proposed, based on the conjecture that miRNAs having similar function are likely to be related to phenotypically similar diseases [23–25]. For instance, Jiang *et al.* [26] developed a network-based computational approach to predict miRNA-disease associations by taking advantage of integration of miRNA functional network, human phenome-miRNAome network, and known miRNA-disease association network. However, the method only adopted local neighbor information, which greatly limited the performance. Moreover, depending on the postulation that target genes will perform abnormal regulation if miRNAs are included in a specific tumor phenotype , Xu *et al.* [27] devised a model according to the miRNA target-dysregulated network (MTDN) to infer new disease related miRNAs. What MTDN differs from other network-based model is that it identified dysregulated network edges (regulations) rather than dysregulated nodes (miRNAs) to assemble disease-related signatures. But the MTDN only focused on prostate cancer, topological feature difference may result in improper outcomes when we applied MTDN to other diseases. Besides, Chen *et al.* [28] developed a method, HGIMDA, integrating various known heterogeneous databases including disease semantic similarity, miRNA functional similarity, Gaussian interaction profile kernel similarity and experimentally validated miRNA-disease associations into a heterogeneous network to identify potential miRNA-disease associations. HGIMDA was developed based on an iterative procedure to figure out the optimal solutions based on the integrated global network, where it inferred possible relationship between certain disease and miRNA by calculating all paths satisfying specific condition. However, the selection of decay factor in the model remains unresolved. Furthermore, Chen *et al.* [29] proposed a model of Within and Between Score for MiRNA-disease Association Prediction (WBSMDA) to predict potential miRNA-disease associations. WBSMDA calculated the Within-Score, finding miRNA achieving highest-similarity-score among miRNAs having relationship with the investigated disease, and Between-Score, finding miRNA achieving highest-similarity-score among miRNAs without the known relationship with the investigated disease, to predict potential miRNA-disease associations. Nevertheless, result of WBSMDA shows that its performance is still not satisfying. By considering information of miRNA cluster and family , Xuan *et al.* [30] devised an approach of Prediction of microRNAs Associated with Human Diseases Based on Weighted *k* Most Similar Neighbors (HDMP). In the framework of HDMP, miRNAs in a cluster or family were assigned higher weight while constructing miRNA functional similarity matrix to further calculate relevance score with investigated disease because of their higher probability to be related with similar diseases. In addition, random walk and its various variants have been broadly applied in bioinformatics, such as disease gene prediction [31], disease-related long non-coding RNAs prediction [32–34], drug-target interaction prediction [35, 36], disease-related miRNA-environmental factor interactions prediction [37] and disease-related microbiota prediction. Consequently, several miRNA-disease association prediction models were designed by implementing random walk to prioritize related miRNAs for diseases. Chen *et al.* [38] presented a method of Random Walk with Restart for MiRNA-Disease Association (RWRMDA) by applying random walk with restart on the miRNA functional similarity network. RWRMDA made full use of global similarity network rather than local one, compared with previous models, which has been proved to improve performance. However, the main defect of the method is that it is not able to predict for diseases any known associated miRNA. Furthermore, another random-walk applied framework focusing on the functional connection between disease genes and miRNA targets in protein-protein interaction (PPI) networks at the systematic level was proposed by Shi *et al.* [39]. For the purpose of identifying the functional links, which was used to construct a bipartite miRNA-disease network, random walk analysis was implemented as a distance measure method. Recently, Xuan *et al.* [40] have also constructed a miRNA-disease association prediction model based on random walk. This model exploited nodes’ prior information and local topological structures of the different classes of nodes by constructing miRNA network based on paired miRNAs’ associated diseases information and assigning different weights to different nodes. Moreover, by involving protein information, Mork *et al.* [41] proposed scoring schemes that ranked miRNA-disease associations by combining protein-disease association scores and miRNA-protein association scores.

Apart from the aforementioned models, computational approaches deploying machine learning methods are becoming increasingly prevailing in bioinformatics [42–44]. Chen *et al.* [45] proposed a model of Regularized Least Squares for MiRNA-Disease Association (RLSMDA) to identify potential association between miRNAs and diseases, which is a global and semi-supervised method without need of negative samples. RLSMDA is capable of predicting potentially associated miRNAs for diseases without known related miRNAs. However, the performance is not satisfactory enough. Furthermore, a computational model of Restricted Boltzmann machine for multiple types of miRNA-disease association prediction (RBMMMDA) was devised in order to discern different miRNA-disease association types [46]. RBM model is a bilayer undirected graphical model including layers of visible modules, disease, and invisible modules, unknown features describing miRNA-disease associations, to predict both the miRNA-disease associations and its corresponding types. Nevertheless, parameter selection remains unresolved in RBMMMDA. In conclusion, previous models have the following limitations. First of all, some methods need negative samples, which is difficult to identify in miRNA-disease association network. Besides, the information provided by the known miRNA, disease and miRNA-disease networks has not been fully exploited. Furthermore, some models rely heavily on parameter selection, which remains unsolved at last. Therefore, a reliable and effective approach for predicting potential miRNA-disease associations is eagerly necessitated. In order to clearly illustrate the input, output and limitation of each computational models aforementioned, we published a comparison table, Supplementary Table 1.

In this study, we proposed a novel computational method of Personalized Recommendation-based MiRNA-Disease Association prediction (PRMDA). Recommendation algorithms, as a universal computational algorithm, has been applied in many aspects including bioinformatics [47]. The reason why we choose personalized recommendation algorithm is that among all recommendation algorithms, personalized recommendation algorithm is remarkably superior in dealing with data sparsity and scalability compared with other algorithms. Generally, in e-commerce system, personalized recommendation algorithm could effectively solve the data sparseness and cold start problems without much participation of users, which corresponds to our study of sparsely distributed data of miRNAs and diseases and prioritizing potentially associated miRNAs for new diseases without known related miRNAs. In our study, potential miRNA-disease associations are recommended with high priority by taking the information of related miRNAs and diseases into account for each miRNA-disease pair respectively, as the name “personalized” suggests, to exploit the similarity network expansively. By integrating known miRNA-disease association network, miRNA-miRNA functional similarity network and disease-disease semantic similarity network to predict potential miRNA-disease associations, PRMDA is a global method that is capable of prioritizing miRNAs for all diseases simultaneously. Besides, PRMDA could prioritize candidate miRNAs for diseases without any known related miRNAs. More importantly, we implemented personalized recommendation-based algorithm on integrated similarity network for miRNAs and diseases, based on miRNA functional similarity network, disease semantic network and Gaussian interaction profile kernel similarity, to remarkably reduce data sparseness. As a result, PRMDA showed superior performance in leave-one-out cross validation by obtaining superior AUC result, which outperformed previous prediction models. Besides, in the case studies of a few important human cancers, more than 80% out of top 50 predicted miRNAs for Colon Neoplasms (CN), Esophageal Neoplasms (EN), Lymphoma, Lung Neoplasms (LN) and Breast Neoplasms (BN) have been experimentally validated. We could draw a conclusion that PRMDA is an efficient and reliable miRNA-disease association prediction model.

## RESULTS

### Performance evaluation

In order to evaluate the prediction accuracy of PRMDA, we implemented leave-one-out cross validation (LOOCV) based on verified miRNA-disease associations recorded in the HMDD V2.0 database [48], along with performance comparison among five advanced computational approaches for miRNA-disease association prediction: HGIMDA [28], RLSMDA [45], HDMP [30], WBSMDA [29], and RWRMDA [38]. For the procedure of LOOCV, each known disease-related miRNA was regarded as a test sample consecutively; and the training set was composed of all other 5429 known miRNA-disease associations. In each turn, a test sample was considered to be a successful prediction if its rank was higher than the given threshold, while compared with all the candidate miRNAs, having no verified association with the investigated disease. As for the disease with only one known related miRNA, which will have no related miRNAs in LOOCV, PRMDA could use constructed global network to infer the potentially associated miRNAs for the disease according to the verified related miRNAs of other similar diseases. In order to better illustrate the performance of PRMDA, Receiver operating characteristics (ROC) curve was drawn by plotting the true positive rate (TPR, sensitivity) against the false positive rate (FPR, 1-specificity) according to different thresholds. Sensitivity measures the ratio of positives that are correctly identified, which denotes the proportion of the test miRNA-disease associations scoring points greater than the assigned threshold in this study. In contrast, specificity measures the ratio of negatives that are correctly identified, which indicates the proportion of negative miRNA-disease pairs scoring ranks less than the given threshold. We calculated Area under the ROC curve (AUC) to evaluate the forecast capability of PRMDA. Here, AUC = 1 suggests perfect prediction performance of the evaluated model, and AUC = 0.5 means that the method makes the prediction randomly.

**Figure 1:**
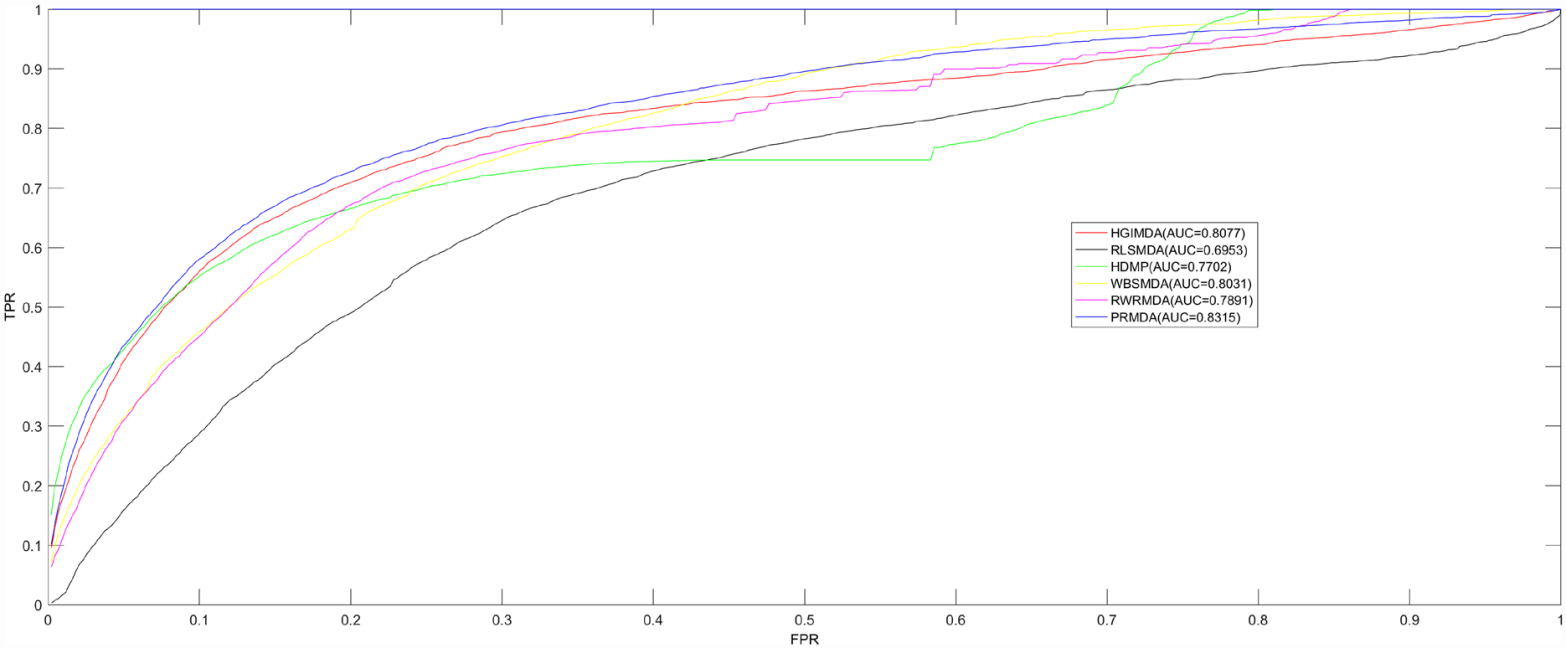
Performance comparisons between PRMDA and five advanced disease-miRNA association prediction models (HGIMDA, RLSMDA, HDMP, WBSMDA, and RWRMDA) in terms of ROC curve and AUC based on the framework of LOOCV As a result, PRMDA achieved AUC of 0.8315 in LOOCV, significantly outperforming all the previous computational models in prediction accuracy.

The performance comparison in terms of LOOCV results was shown in Figure 1. As a result, in the LOOCV, PRMDA, HGIMDA, RLSMDA, HDMP, WBSMDA, and RWRMDA achieved AUCs of 0.8315, 0.8077, 0.6953, 0.7702, 0.8031, and 0.7891, correspondingly. It is apparent that the performance of PRMDA outperformed previous prediction models to a great degree based on known miRNA-disease associations. We can draw a conclusion that PRMDA has displayed accurate and credible prediction performance and possesses the practical value to uncover unknown miRNA-disease associations.

### Case studies

In order to further demonstrate the reliability precision of PRMDA, we carried out case studies of several vital human cancers. Prediction results were confirmed by matching miRNA-disease associations verified by experimental reports to another two databases: miR2Disease [49] and dbDEMC [50]. We implemented three kinds of case studies in all. Firstly, for case studies of CN, EN and Lymphoma, we implemented PRMDA for prediction on all miRNA-disease associations recorded in the HMDD V2.0 database. In the second type of case study for LN, we removed all known related miRNAs with LN and then implemented PRDMA to infer potential related miRNAs for LN, which means that PRDNA could also work for diseases having no related miRNA. As for the case study of BN, we applied PRMDA to identify potential miRNA-disease associations based on the HMDD V1.0 database and matched the results with the data in miR2Disease, dbDEMC and HMDD V2.0.

As the most common type of gastrointestinal cancer, CN, poses great threaten to human’s lives [51, 52]. Owing to the intricacy of taking precautions against metastatic disease with apposite therapies, statistics indicate that half of the patients suffering from CN die of metastatic disease within 5 years after being diagnosed [53]. In the past few years, researchers managed to identify several related miRNAs for CN. For instance, Guo *et al.* found that an omnipresent absence of miR-126 in CN lines in comparison with normal human colon epithelia. Consequently, the experimental evidence proved that the down-regulation of miR-126 weakens its function as growth suppressor in CN cells, which indirectly promotes CN development [54]. The miRNA hsa-mir-145 down-regulates the insulin receptor substrate-1 (IRS-1), an abutting protein for receptors, and inhibits the growth of CN cells [55]. In our case study of CN, PRMDA was implemented to select the highest-rank miRNAs from candidate miRNAs for CN (See Table 1).The result suggests that all of the top ten candidate miRNAs have been confirmed to be related to CN. Besides, 92% of top 50 prioritized miRNAs were confirmed to have association with CN. Taking miRNA has-mir-21 (rated 1st in prediction list) for example, numerous experiments validated the significantly higher expression of has-mir-21 in CN pathological tissue than adjacent common tissue [56, 57]. In addition, studies also confirmed that high expression of hsa-mir-155, ranked 2nd in prediction list, was closely correlated with lymph node metastases, which promoted CN tumor growth [58]. The miRNA hsa-let-7a, ranked 3rd in the list, was detected to perform down-regulation in clinical experiment for CN patients [59].

**Table 1:** Prediction list of the top 50 prioritized miRNAs associated with Colon Neoplasms based on known associations in HMDD V2.0 database

miRNA	Evidence	Score	miRNA	Evidence	Score
hsa-mir-21	dbDEMC, miR2Disease	0.403491	hsa-let-7d	dbDEMC	0.331462
hsa-mir-155	dbDEMC, miR2Disease	0.392804	hsa-mir-127	dbDEMC, miR2Disease	0.326204
hsa-let-7a	dbDEMC, miR2Disease	0.389646	hsa-mir-29a	dbDEMC, miR2Disease	0.324617
hsa-mir-34a	dbDEMC, miR2Disease	0.37374	hsa-mir-146a	dbDEMC	0.323809
hsa-mir-20a	dbDEMC, miR2Disease	0.373494	hsa-mir-221	dbDEMC, miR2Disease	0.32359
hsa-mir-200b	dbDEMC	0.371881	hsa-let-7g	dbDEMC, miR2Disease	0.321759
hsa-let-7b	dbDEMC, miR2Disease	0.371038	hsa-mir-34c	miR2Disease	0.319629
hsa-mir-125b	dbDEMC	0.368865	hsa-mir-191	dbDEMC, miR2Disease	0.318579
hsa-let-7c	dbDEMC	0.363012	hsa-mir-218	dbDEMC	0.316699
hsa-mir-18a	dbDEMC, miR2Disease	0.36237	hsa-mir-9	dbDEMC, miR2Disease	0.311736
hsa-mir-143	dbDEMC, miR2Disease	0.359626	hsa-mir-10b	dbDEMC, miR2Disease	0.310447
hsa-mir-200c	dbDEMC, miR2Disease	0.357541	hsa-mir-34b	dbDEMC, miR2Disease	0.309224
hsa-mir-19a	dbDEMC, miR2Disease	0.352472	hsa-mir-25	dbDEMC, miR2Disease	0.309005
hsa-mir-16	dbDEMC	0.35214	hsa-mir-132	miR2Disease	0.308697
hsa-let-7e	dbDEMC	0.35181	hsa-mir-30c	dbDEMC, miR2Disease	0.306546
hsa-mir-19b	dbDEMC, miR2Disease	0.34718	hsa-mir-106b	dbDEMC, miR2Disease	0.305881
hsa-mir-141	dbDEMC, miR2Disease	0.338829	hsa-mir-29b	dbDEMC, miR2Disease	0.30437
hsa-mir-92a	unconfirmed	0.338446	hsa-mir-196a	dbDEMC, miR2Disease	0.301877
hsa-let-7f	dbDEMC, miR2Disease	0.33778	hsa-mir-429	dbDEMC	0.301279
hsa-mir-101	unconfirmed	0.336823	hsa-mir-222	dbDEMC	0.301203
hsa-mir-223	dbDEMC, miR2Disease	0.336049	hsa-mir-1	dbDEMC, miR2Disease	0.300921
hsa-mir-199a	unconfirmed	0.335912	hsa-mir-205	dbDEMC	0.298187
hsa-mir-200a	unconfirmed	0.335622	hsa-mir-192	dbDEMC, miR2Disease	0.296645
hsa-let-7i	dbDEMC	0.333927	hsa-mir-107	dbDEMC, miR2Disease	0.296079
hsa-mir-125a	dbDEMC, miR2Disease	0.333203	hsa-mir-210	dbDEMC	0.2941

Regarded as one of the most common cancer worldwide, EN is typically diagnosed at a partially advanced phase or at a phase involving lymph nodes. Besides, the general five-year survival rate of EN keeps at a low level, which requires novel miRNA-disease prediction for disease detection at an early stage. According to previous studies, miRNA deregulation are frequently detected in EN, suggesting that miRNAs are of great significance to tumorigenesis [60]. For instance, studies found that Notch-1 specific miRNAs miR-21 and miR-34a are down-regulated during curcumin, a powerful inhibitor of EN growth, treatment [61]. And the upregulated expression of tumor suppressor let-7a is an extremely important determining factor in reacting to chemotherapy by regulating IL-6/STAT3 pathway in esophageal squamous cell carcinoma [62]. In our case study of EN by implementing PRMDA (See Table 2), 8 out of the top 10 predicted miRNAs have been validated to be EN-related by dbDEMC and miR2Disease datasets. Furthermore, 94% of the top 50 candidate miRNAs were verified.

**Table 2:** Prediction list of the top 50 prioritized miRNAs associated with Esophageal Neoplasms based on known associations in HMDD V2.0 database

miRNA	Evidence	Score	miRNA	Evidence	Score
hsa-mir-200b	dbDEMC	0.354477	hsa-mir-106a	dbDEMC	0.288761
hsa-mir-17	dbDEMC	0.341267	hsa-mir-93	dbDEMC	0.288345
hsa-let-7f	unconfirmed	0.335941	hsa-mir-191	dbDEMC	0.28728
hsa-let-7e	dbDEMC	0.335759	hsa-mir-146b	dbDEMC	0.286041
hsa-mir-18a	dbDEMC	0.334254	hsa-mir-302b	dbDEMC	0.281647
hsa-mir-125b	dbDEMC	0.334037	hsa-mir-132	dbDEMC	0.28155
hsa-let-7d	dbDEMC	0.329478	hsa-mir-302c	dbDEMC	0.281246
hsa-mir-218	unconfirmed	0.324689	hsa-mir-142	dbDEMC	0.27999
hsa-let-7i	dbDEMC	0.3231	hsa-mir-29b	dbDEMC	0.279844
hsa-mir-10b	dbDEMC	0.32263	hsa-mir-199b	dbDEMC	0.273617
hsa-mir-16	dbDEMC	0.31715	hsa-mir-107	dbDEMC, miR2Disease	0.27071
hsa-mir-429	dbDEMC	0.31628	hsa-mir-181a	dbDEMC	0.268884
hsa-let-7g	dbDEMC	0.314976	hsa-mir-24	dbDEMC	0.268299
hsa-mir-19b	dbDEMC	0.313096	hsa-mir-30a	dbDEMC	0.266871
hsa-mir-125a	dbDEMC	0.312709	hsa-mir-181b	dbDEMC	0.265328
hsa-mir-9	dbDEMC	0.30476	hsa-mir-194	dbDEMC, miR2Disease	0.264892
hsa-mir-221	dbDEMC	0.304585	hsa-mir-182	dbDEMC	0.263903
hsa-mir-29a	dbDEMC	0.302556	hsa-mir-20b	dbDEMC	0.263734
hsa-mir-1	dbDEMC	0.297728	hsa-mir-195	dbDEMC	0.262951
hsa-mir-127	dbDEMC	0.297439	hsa-mir-30d	dbDEMC	0.26244
hsa-mir-222	dbDEMC	0.297081	hsa-mir-204	unconfirmed	0.262031
hsa-mir-106b	dbDEMC	0.296993	hsa-mir-373	dbDEMC, miR2Disease	0.256662
hsa-mir-7	dbDEMC	0.296578	hsa-mir-372	dbDEMC	0.254717
hsa-mir-18b	dbDEMC	0.291515	hsa-mir-15b	dbDEMC	0.250247
hsa-mir-30c	dbDEMC	0.289068	hsa-mir-367	dbDEMC	0.249998

Lymphomas are always classified into two types: Hodgkin Lymphomas (HL) and non-Hodgkin Lymphomas (NHL). HL, which is far more common than NHL, derives from preapoptotic germinal center B cells, where universal deficiency of B cell phenotype is distinguished [63]. NHL is treated mostly through local radiotherapy and chemotherapy treatment [64]. Recent studies found that PRDM1/blimp-1, a major regulator in terminal B-cell differentiation, is also a target for down-regulation mediated by miR-9 and let-7a in HL cell line, which functionally targeted specific pairing position in the PRDM1/blimp-1 mRNA 3′ untranslated region and suppressed luciferase reporter liveness by repressing translation [65]. Besides, researchers also discovered that the distinct set of five miRNAs (miR-150, miR-550, miR-518b, miR-124a and miR-539) was differentially expressed in gastritis in contrast with MALT lymphoma [66]. We implemented PRMDA on HMDD V2.0 database to prioritize related miRNAs for Lymphoma (See Table 3). As a result, 9 out of the top 10 candidate miRNAs for Lymphoma and 43 out of the top 50 miRNAs in the prediction list have been verified by the researches recorded in dbDEMC and miR2Disease databases.

**Table 3:** Prediction list of the top 50 prioritized miRNAs associated with Lymphoma based on known associations in HMDD V2.0 database

miRNA	Evidence	Score	miRNA	Evidence	Score
hsa-mir-34a	dbDEMC	0.394951	hsa-mir-10b	dbDEMC	0.28162
hsa-mir-125b	unconfirmed	0.392223	hsa-mir-181b	dbDEMC	0.278209
hsa-let-7a	dbDEMC	0.362083	hsa-mir-429	unconfirmed	0.277427
hsa-mir-145	dbDEMC, miR2Disease	0.351275	hsa-mir-182	dbDEMC	0.276958
hsa-mir-221	dbDEMC, miR2Disease	0.34684	hsa-mir-142	unconfirmed	0.275153
hsa-mir-223	dbDEMC	0.344073	hsa-mir-195	dbDEMC	0.273455
hsa-let-7d	dbDEMC	0.329285	hsa-mir-146b	unconfirmed	0.273431
hsa-let-7b	dbDEMC	0.327671	hsa-mir-27a	dbDEMC	0.273084
hsa-mir-9	dbDEMC	0.318525	hsa-mir-106a	dbDEMC, miR2Disease	0.273011
hsa-let-7c	dbDEMC	0.317094	hsa-mir-25	dbDEMC	0.272812
hsa-mir-29b	dbDEMC	0.31624	hsa-mir-127	dbDEMC, miR2Disease	0.270926
hsa-mir-106b	dbDEMC	0.315872	hsa-mir-141	dbDEMC	0.26861
hsa-let-7f	dbDEMC	0.314598	hsa-mir-7	dbDEMC	0.260548
hsa-mir-222	dbDEMC	0.313115	hsa-mir-30e	dbDEMC	0.259788
hsa-let-7e	dbDEMC, miR2Disease	0.307227	hsa-mir-373	dbDEMC	0.255789
hsa-mir-29a	dbDEMC	0.303588	hsa-mir-183	unconfirmed	0.255254
hsa-let-7i	dbDEMC	0.300001	hsa-mir-302b	unconfirmed	0.254974
hsa-mir-205	dbDEMC	0.29947	hsa-mir-339	dbDEMC	0.254438
hsa-mir-34b	dbDEMC	0.297126	hsa-mir-30d	dbDEMC	0.253573
hsa-mir-93	dbDEMC	0.293033	hsa-mir-30c	dbDEMC	0.253055
hsa-mir-143	dbDEMC, miR2Disease	0.292999	hsa-mir-191	dbDEMC	0.251247
hsa-mir-199a	dbDEMC	0.291715	hsa-mir-30a	dbDEMC	0.250674
hsa-let-7g	dbDEMC	0.287751	hsa-mir-192	dbDEMC	0.249858
hsa-mir-214	dbDEMC	0.287659	hsa-mir-302c	dbDEMC	0.249705
hsa-mir-34c	unconfirmed	0.285456	hsa-mir-148a	dbDEMC	0.248657

We further compared PRMDA with another three recent miRNA-disease association prediction models, MCMDA [67], HGIMDA [28] and WBSMDA [29], in terms of the case studies of CN, EN and Lymphoma. The comparison was presented in Table 4. Besides, we conducted related-miRNA prediction and verification for more diseases, and the number of verified miRNA-disease associations are presented in Table 5.

**Table 4:** Comparison between PRMDA, MCMDA, HGIMDA and WBSMDA of the case studies

Disease	PRMDA	MCMDA	HGIMDA	WBSMDA
Colon Neoplasms	46	42	45	45
Esophageal Neoplasms	47	31	44	29
Lymphoma	44	39	45	42

The second, third, fourth and fifth column record the number of verified miRNA-diseases associations among top 50 prioritized associations by PRMDA, MCMDA, HGIMDA and WBSMDA respectively.

**Table 5:** Prediction results of several human diseases

Disease	TOP 10	TOP 20	TOP 50
Glioblastoma	1	10	27
Lung Neoplasms	8	15	31
Colonic Neoplasms	8	15	38
Breast Neoplasms	8	15	40
Prostate Neoplasms	9	18	43
Kidney Neoplasms	8	17	44
Colon Neoplasms	10	18	46
Esophageal Neoplasms	8	18	47

The second, third and fourth column record the number of verified miRNA-disease associations based on known associations in HMDD V2.0 database out of top 10, top 20 and top 50 respectively.

LN is considered to be responsible for considerable mortality worldwide. According to statistics, compared with never smokers, former smokers are far more likely to come down with LN [68]. Currently, diagnosing LN at an early stage remains difficult for the majority, meanwhile five-year survival rates are less than 15% after diagnosis. Recent studies suggested that using miRNA-related methods for LN detection could be more effective than the main detecting method: screening computed tomography scans [69]. Many LN-related miRNAs were identified by researchers in the past few years. For instance, Takamizawa *et al.* issued the first report of reduced expression of miRNA let-7 in human LN and suggested the potential clinical and biological influence of miRNA dysfunction [70]. Besides, miRNAs miR-511 and miR-1297 act as LN tumor suppressor genes, which could suppress adenocarcinoma cell proliferation *in vitro* and *in vivo* by indirectly increasing CCAAT/enhancer-binding protein alpha expression [71]. Here, we implemented the second type of case study on LN by removing all known miRNA-LN associations (See Table 6). All of the top 10 and 48 out of the top 50 prioritized miRNAs have been verified to be related to LN by experiments. In this way, PRMDA presented excellent prediction capability for diseases without known related miRNAs.

**Table 6:** Prediction list of the top 50 prioritized miRNAs associated with Lung Neoplasms by removing all known miRNAs related with Lung Neoplasms in HMDD V2.0 database

miRNA	Evidence	Score	miRNA	Evidence	Score
hsa-mir-21	dbDEMC,miR2Disease,HMDD	0.392142	hsa-mir-199a	dbDEMC,miR2Disease,HMDD	0.332737
hsa-let-7a	dbDEMC,miR2Disease,HMDD	0.383318	hsa-mir-125a	dbDEMC,miR2Disease,HMDD	0.330086
hsa-mir-145	dbDEMC,miR2Disease,HMDD	0.377187	hsa-mir-9	miR2Disease,HMDD	0.329039
hsa-mir-34a	dbDEMC	0.376841	hsa-mir-93	dbDEMC,miR2Disease,HMDD	0.324393
hsa-let-7b	miR2Disease,HMDD	0.372622	hsa-mir-101	dbDEMC,miR2Disease,HMDD	0.323704
hsa-let-7d	dbDEMC,miR2Disease,HMDD	0.365874	hsa-mir-210	dbDEMC,miR2Disease,HMDD	0.321786
hsa-mir-125b	miR2Disease HMDD	0.364603	hsa-mir-223	unconfirmed	0.319515
hsa-mir-155	dbDEMC,miR2Disease,HMDD	0.362776	hsa-mir-200a	dbDEMC,miR2Disease,HMDD	0.318552
hsa-mir-17	miR2Disease,HMDD	0.358777	hsa-mir-7	miR2Disease,HMDD	0.318257
hsa-let-7c	dbDEMC,miR2Disease,HMDD	0.355499	hsa-mir-92a	unconfirmed	0.318223
hsa-mir-34b	dbDEMC	0.352716	hsa-mir-10b	dbDEMC	0.31628
hsa-let-7e	miR2Disease HMDD	0.352674	hsa-mir-221	dbDEMC	0.315655
hsa-mir-34c	dbDEMC	0.348275	hsa-mir-214	dbDEMC,miR2Disease,HMDD	0.315203
hsa-mir-200c	dbDEMC,miR2Disease,HMDD	0.348273	hsa-mir-196a	dbDEMC	0.31437
hsa-mir-126	dbDEMC,miR2Disease,HMDD	0.347795	hsa-mir-1	dbDEMC,miR2Disease,HMDD	0.313729
hsa-let-7f	miR2Disease HMDD	0.34451	hsa-mir-146b	miR2Disease HMDD	0.313125
hsa-mir-205	dbDEMC,miR2Disease,HMDD	0.343288	hsa-mir-27a	dbDEMC	0.312186
hsa-mir-18a	dbDEMC,miR2Disease,HMDD	0.342298	hsa-mir-146a	dbDEMC,miR2Disease,HMDD	0.311986
hsa-mir-200b	dbDEMC,miR2Disease,HMDD	0.342297	hsa-mir-143	dbDEMC,miR2Disease,HMDD	0.30695
hsa-mir-20a	dbDEMC,miR2Disease,HMDD	0.341235	hsa-mir-25	dbDEMC	0.306837
hsa-mir-218	dbDEMC,miR2Disease,HMDD	0.341204	hsa-mir-29b	dbDEMC,miR2Disease,HMDD	0.306497
hsa-let-7g	dbDEMC,miR2Disease,HMDD	0.339591	hsa-mir-16	dbDEMC,miR2Disease,HMDD	0.302916
hsa-let-7i	dbDEMC	0.33887	hsa-mir-222	dbDEMC	0.301147
hsa-mir-19b	dbDEMC	0.337602	hsa-mir-30d	dbDEMC	0.301058
hsa-mir-19a	dbDEMC,miR2Disease,HMDD	0.336874	hsa-mir-29a	dbDEMC,miR2Disease,HMDD	0.301033

BN contributes most to cancer-caused deaths in women at the age of 40 and younger in developed countries [72]. Worse still, the survival rates of young women with BN remain lower than those of elder women [73]. Therefore, it is an increasingly urgent problem in low- and middle-income countries all over the world [74]. It has been acknowledged that gene-expression profiling exerts substantial influence on our comprehending of BN biology. In the past two decades, f innate molecular subclasses of BN (Luminal A, Luminal B, HER2-enriched, Basal-like and Claud in-low) have been discovered and intensively studied [75]. A typical example is that miRNA let-7a represses BN cell migration and invasion through downregulation of C-C chemokine receptor type 7, which is critical in metastatic and chemotactic responses in numerous cancers including BN [76]. Here, in order to evaluate the performance, we also implemented PRMDA on the HMDD V1.0 database to predict candidate miRNAs for BN (See Table 7). As the result suggests, 100% of the top 10 candidate related miRNAs are proved to be BN-associated, and 44 out of the top 50 rated miRNAs are verified to be related with BN by HMDD V2.0, dbDEMC and miR2Disease datasets.

**Table 7:** Prediction list of the top 50 prioritized miRNAs associated with Breast Neoplasms based on known associations in HMDD V1.0 database

miRNA	Evidence	Score	miRNA	Evidence	Score
hsa-let-7e	dbDEMC, HMDD	0.342426	hsa-mir-99b	dbDEMC	0.215248
hsa-let-7b	dbDEMC, HMDD	0.331861	hsa-mir-30e	unconfirmed	0.214727
hsa-let-7i	dbDEMC, miR2Disease,HMDD	0.325423	hsa-mir-32	dbDEMC	0.213762
hsa-let-7c	dbDEMC, HMDD	0.317913	hsa-mir-611	unconfirmed	0.212971
hsa-mir-16	dbDEMC, HMDD	0.313056	hsa-mir-583	dbDEMC	0.212971
hsa-mir-92a	HMDD	0.304199	hsa-mir-602	dbDEMC	0.212971
hsa-let-7g	dbDEMC, HMDD	0.301935	hsa-mir-615	dbDEMC	0.212971
hsa-mir-223	dbDEMC, HMDD	0.29829	hsa-mir-654	dbDEMC	0.212971
hsa-mir-191	dbDEMC, miR2Disease,HMDD	0.272251	hsa-mir-486	dbDEMC, HMDD	0.212971
hsa-mir-126	dbDEMC, miR2Disease,HMDD	0.271694	hsa-mir-769	unconfirmed	0.212971
hsa-mir-101	dbDEMC,miR2Disease,HMDD	0.268627	hsa-mir-557	dbDEMC	0.212971
hsa-mir-18b	dbDEMC, HMDD	0.255543	hsa-mir-601	dbDEMC	0.212971
hsa-mir-106a	dbDEMC	0.2538	hsa-mir-642	unconfirmed	0.212971
hsa-mir-92b	dbDEMC	0.251822	hsa-mir-518c	dbDEMC	0.212971
hsa-mir-181a	dbDEMC, miR2Disease,HMDD	0.241862	hsa-mir-324	HMDD	0.212971
hsa-mir-373	dbDEMC, miR2Disease,HMDD	0.237108	hsa-mir-608	dbDEMC, HMDD	0.212971
hsa-mir-29c	dbDEMC, miR2Disease,HMDD	0.236599	hsa-mir-662	dbDEMC	0.212971
hsa-mir-203	dbDEMC, miR2Disease,HMDD	0.231891	hsa-mir-596	unconfirmed	0.212971
hsa-mir-142	Unconfirmed	0.226825	hsa-mir-185	dbDEMC	0.212971
hsa-mir-130b	dbDEMC	0.219054	hsa-mir-600	dbDEMC	0.212971
hsa-mir-24	dbDEMC, HMDD	0.218864	hsa-mir-622	dbDEMC	0.212971
hsa-mir-15b	dbDEMC	0.218328	hsa-mir-629	dbDEMC, HMDD	0.212971
hsa-mir-575	dbDEMC	0.217562	hsa-mir-638	dbDEMC, HMDD	0.212971
hsa-mir-128b	miR2Disease	0.217551	hsa-mir-637	dbDEMC	0.212971
hsa-mir-197	dbDEMC, HMDD	0.217169	hsa-mir-612	dbDEMC	0.212971

The results of case studies and cross validation strongly indicate that PRMDA is a reliable and effective computational model for potential miRNA-disease association prediction referring to known associations. At last, we further applied PRMDA to predict related miRNAs for every disease and published the prediction lists of miRNA and disease pairs in supplementary table (See Supplementary Table 2) based on known miRNA-disease associations recorded in HMDD V2.0. The predicted pairs with higher ranks could be given reasonable priority for future researches. We also provided the score ranked by all miRNA-disease association pairs for each disease in Supplementary File 1.

## DISCUSSION

The prominent performance of PRMDA could be attributed to several following factors. First of all, PRMDA implemented personalized recommendation-based algorithm on integrated similarity for both miRNAs and diseases. Moreover, as one of the most prevailing recommendation algorithm, personalized recommendation-based algorithm could remarkably improve data sparseness especially for diseases and miRNAs with few known related miRNAs and diseases, in which the content of associated miRNAs and diseases are taken into consideration for each miRNA-disease pair respectively, as the name “personalized” implies, to utilize the similarity network expansively. Secondly, PRMDA is a global method, which could prioritize miRNAs for all diseases simultaneously. Compared with the previous model, the significantly increasing amount of verified miRNA-disease associations involved in our model further ensures the credibility of prediction results. Last of all, the success of PRMDA gives credit to integration of miRNA-disease association network, disease semantic similarity, miRNA functional similarity, and Gaussian interaction profile kernel similarity, which promotes the precision and diminishes bias caused by incomplete database simultaneously during prediction. Furthermore, Sun et al. [77] proposed a model of MiRNA-Disease Association based on Network Topological Similarity (NTMSDA). NTMSDA constructed 2 novel adjacent matrixes according to miRNA and disease network topological similarity matrix. Nevertheless, NTMSDA failed to prioritize miRNAs for diseases without known related miRNAs due to its strict topological dependence. NTMSDA is also a recommendation-based method, the difference from PRMDA lies in several aspects: firstly, the way building new rating matrix for miRNAs and diseases was totally different. Secondly, NTMSDA requires parameter selection during the process of incorporating the two new integrated adjacent matrixes. Lastly, compared with NTSMDA, PRMDA integrated more similarity information in the last step of ranking. With more and more discoveries of new diseases, which do not have related miRNAs, PRMDA could perfectly work for such diseases, as well as prioritizing diseases for newly identified miRNAs with no related diseases.

However, there are still some existing limitations that could be ameliorated in the future. Firstly, the completeness of miRNA-disease association network remains to be enriched with more experimental validations. Secondly, the performance of PRMDA may be improved by integrating more datasets which provide other information about miRNAs, diseases and associations between them. Finally, the personalized recommendation-based algorithm implemented by PRMDA may cause some bias for diseases with more related miRNAs, based on the hypothesis that miRNAs performing function similarly are more probable to be interacted with diseases with similar phenotypes.

## MATERIALS AND METHODS

### Human miRNA-disease associations

Human miRNA-disease associations investigated in PRMDA were downloaded from the HMDD V2.0 database (http://www.cuilab.cn/files/images/hmdd2/alldata.txt) containing 5430 human miRNA-diseases associations confirmed by experimental reports, 383 diseases, and 495 miRNAs. To better demonstrate known miRNA-disease associations, we denoted the association network by the adjacency matrix *A*, where the entity *A (i, j)* equals 1 if there is supporting experimental evidence that miRNA *m(j)* is related to disease *d(i)*, otherwise it equals 0. Besides, we used *n*_m_ and *n*_d_ to represent the number of miRNAs and diseases involved in this study.

### MiRNA functional similarity

MiRNA functional similarity was calculated by MISIM [25], which was composed of four procedures: identifying miRNA-related diseases, calculating sematic values of diseases, calculating sematic similarity for disease pairs and determining miRNA functional similarity based on sematic similarity of related diseases. We downloaded miRNA functional similarity scores from http://www.cuilab.cn/files/images/cuilab/misim.zip in January 2010. Similarly, we built the miRNA functional similarity matrix *MS*, in which the entity *MS (m(i), m(j))* indicates the functional similarity between miRNA *m(i)* and *m(j)*.

### Disease semantic similarity model 1

We downloaded MeSH descriptors from the National Library of Medicine (http://www.nlm.nih.gov/) to construct disease semantic similarity model. Disease-disease associations were depicted into a Directed Acyclic Graph (*DAG*). *DAG (D) = (D,T(D),E(D))* represents the disease *D*, where *T(D)* is the node set containing node *D’s* ancestor nodes and itself, and *E(D)* denotes the corresponding edge set comprising the direct edges from parent nodes to child nodes. The semantic value of disease *D* is calculated as follows:

$$SV_{1}\left(D\right)=\sum_{d\in T\left(D\right)}D1_{D}\left(d\right)$$ (1)

$$\{\begin{array}{ll} D1_{D}\left(d\right)=1 & if\;d=D\\ D1_{D}\left(d\right)=\max\left\{\Delta_{*}D_{D}\left(d^{\prime }\right)|d^{\prime }\;\in \;children\;of\;d\right\} & if\;d\neq D \end{array}$$ (2)

where ∆ is defined as the semantic contribution factor. For a specific disease *D*, the contribution of *D* to the semantic value of disease *D* is considered to equal 1. Furthermore, the contribution is inversely proportional to the distance between *D* and other diseases. Consequently, disease nodes in the same level are believed to contribute the same to the semantic value of disease *D*.

Based on the assumption that larger shared part between two diseases in DAGs indicates larger semantic similarity, we used *DS* to represent the disease semantic similarity matrix, where the semantic similarity between diseases *d (i)* and *d (j)* was calculated as follows:

$$DS_{1}\left(d\left(i\right),d\left(j\right)\right)=\frac{\sum_{t\in T\left(d\left(i\right)\right)\cap T\left(d\left(j\right)\right)}\left(D1_{d\left(i\right)}\left(t\right)+D1_{d\left(j\right)}\left(t\right)\right)}{SV_{1}\left(d\left(i\right)\right)+SV_{1}\left(d\left(j\right)\right)}$$ (3)

The disease semantic matrix calculated by the model 1 is provided in Supplementary Table 3.

### Disease semantic similarity model 2

Here, we also calculated disease sematic similarity in another way. Since diseases in the same level of *DAG(D)* may appear different times in the other disease *DAGs*, the disease semantic similarity model 1 may result in bias of assigning the same contribution for all diseases in the same level. We could conclude that the disease appearing in less disease *DAGs* is more specific and should be given higher contribution.

Here, we defined the contribution of disease *d* to the semantic value of disease *D* in *DAG(D)* as follows.

$$SV_{2}\left(D\right)=\sum_{d\in T\left(D\right)}D2_{D}\left(d\right)$$ (4)

$$\{\begin{array}{ll} D2_{D}\left(d\right)=1 & if\;d=D\\ D2_{D}\left(d\right)=-\log\left(\frac{n_{dt}}{n_{d}}\right) & if\;d\neq D \end{array}$$ (5)

where *n*_*dt*_ is the number of *DAGs* containing disease *d*.

Similarly, we calculated the semantic similarity between diseases *d (i)* and *d (j)* was calculated as follows:

$$DS_{2}\left(d\left(i\right),d\left(j\right)\right)=\frac{\sum_{t\in T\left(d\left(i\right)\right)\cap T\left(d\left(j\right)\right)}\left(D2_{d\left(i\right)}\left(t\right)+D2_{d\left(j\right)}\left(t\right)\right)}{SV_{2}\left(d\left(i\right)\right)+SV_{2}\left(d\left(j\right)\right)}$$ (6)

The disease semantic matrix calculated by the model 2 is provided in Supplementary Table 4.

### Gaussian interaction profile kernel similarity for diseases

Based on the assumption that functionally similar miRNAs are more likely to be associated with phenotypically similar diseases, Gaussian interaction profile kernel similarity for diseases was calculated by taking into consideration the topologic information of known miRNA-disease association network. Firstly, we denoted the interaction profiles of disease *d(i)* with a binary vector *IP(d(i))* by checking whether disease *d(i)* is associated with each miRNA or not, that’s to say, the value of *i*th row in association adjacency matrix *A*. Then, based on the interaction profiles, Gaussian kernel similarity between disease *d(i)* and *d(j)* was calculated as follows:

$$KD\left(d\left(i\right),d\left(j\right)\right)=\exp\left(-\gamma_{d}{\left\|IP\left(d\left(i\right)\right)-IP\left(d\left(j\right)\right)\right\|}^{2}\right)$$ (7)

where the parameter *γ*_*d*_ controls the kernel bandwidth and is obtained through the normalization of a new bandwidth parameter *γ*′_*d*_ by the average number of known related miRNAs for all the diseases.

Thus, *γ*_*d*_ was defined as follows:

$$\gamma_{d}=\gamma_{d}^{\prime }/\left(\frac{1}{n_{d}}\sum_{i=1}^{n_{d}}{\left\|IP\left(d\left(i\right)\right)\right\|}^{2}\right)$$ (8)

At last, *KD* was the Gaussian interaction profile kernel similarity matrix for diseases, where the entity *KD(d(i),d( j))* was the Gaussian interaction profile kernel similarity between disease *d(i)* and disease *d(j)*.

### Gaussian interaction profile kernel similarity for miRNAs

Similar to Gaussian interaction profile kernel similarity calculation for diseases, miRNA Gaussian interaction profile kernel similarity matrix could be calculated in a similar way:

$$KR\left(m\left(i\right),m\left(j\right)\right)=\exp\left(-\gamma_{m}{\left\|IP\left(m\left(i\right)\right)-IP\left(m\left(j\right)\right)\right\|}^{2}\right)$$ (9)

$$\gamma_{m}=\gamma_{m}^{\prime }/\left(\frac{1}{n_{m}}\sum_{i=1}^{n_{m}}{\left\|IP\left(m\left(i\right)\right)\right\|}^{2}\right)$$ (10)

In the equations above, the interaction profile *IP*(*m*(*i*)) for miRNA (*m*(*i*) is determined by whether miRNA *m*(i) is associated with each disease or not. γ_m_ was obtained by normalizing a new bandwidth parameter *γ′*_m_ by the average number of associated diseases for all the miRNAs.

### Integrated similarity for miRNAs and diseases

Integrated miRNA similarity matrix *Sm* and integrated disease similarity matrix *Sd* were established by combining miRNA functional similarity, disease semantic similarity, and Gaussian interaction profile kernel similarity, respectively. The similarity matrix was defined as below:

$$S_{m}\left(m\left(i\right),m\left(j\right)\right)=\{\begin{array}{cc} RS\left(m\left(i\right),m\left(j\right)\right) & m\left(i\right)and\;m\left(j\right)has\;functional\;similarity\\ KR\left(m\left(i\right),m\left(j\right)\right) & otherwise \end{array}$$ (11)

$$S_{d}\left(d\left(i\right),d\left(j\right)\right)=\{\begin{array}{rr} DS\left(d\left(i\right),d\left(j\right)\right) & d\left(i\right)and\;d\left(j\right)has\;semantic\;similarity\\ KD\left(d\left(i\right),d\left(j\right)\right) & otherwise \end{array}$$ (12)

where the semantic similarity between disease *d(i)* and disease *d(j)* was calculated as follows:

$$DS\left(d\left(i\right),d\left(j\right)\right)=\frac{DS_{1}\left(d\left(i\right),d\left(j\right)\right)+DS_{2}\left(d\left(i\right),d\left(j\right)\right)}{2}$$ (13)

### PRMDA

**Figure 2:**
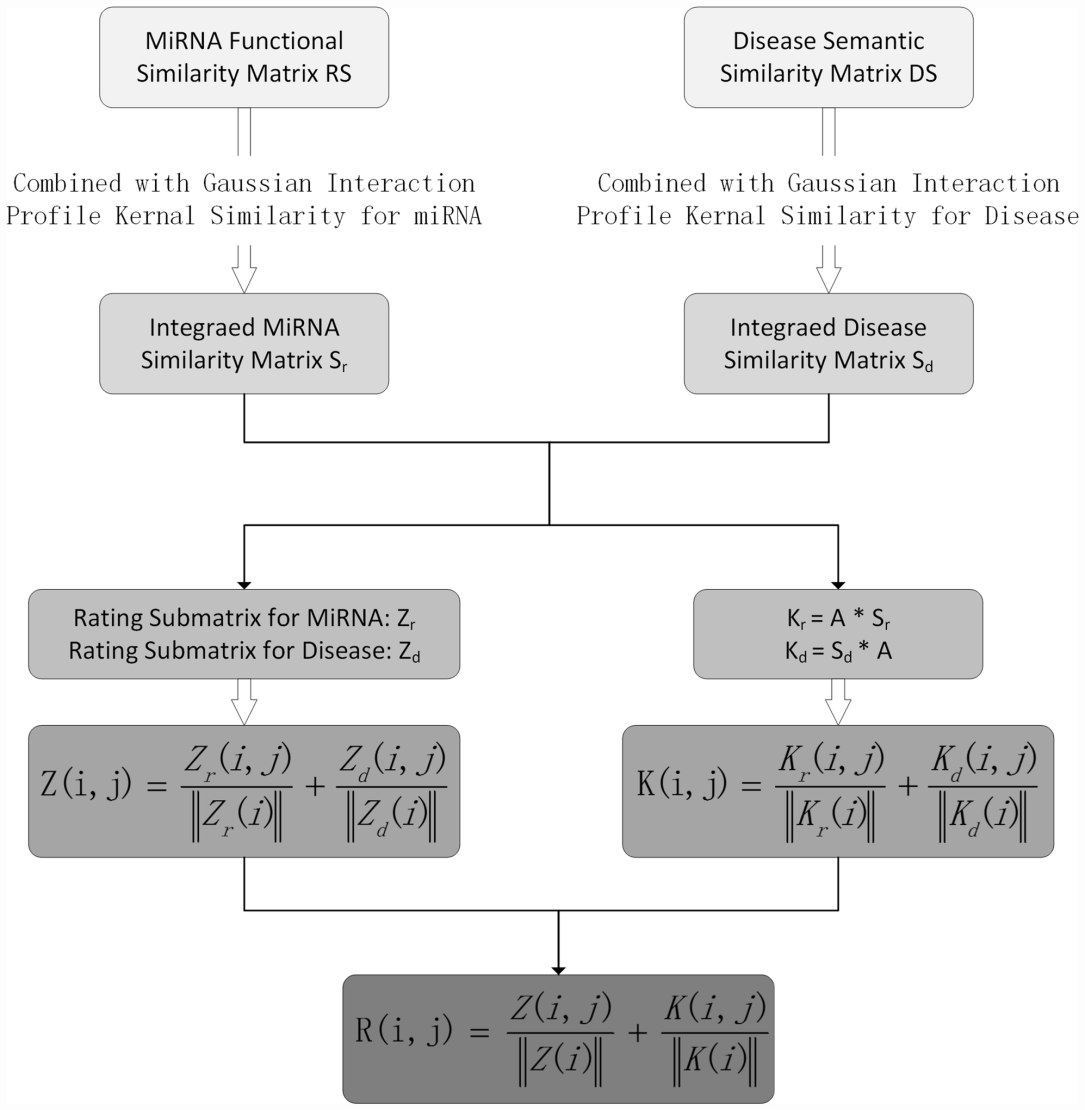
Flowchart of PRMDA model to prioritize potential related miRNA for diseases based on the HMDD V2.0 database

In this study, we proposed a novel computational model of personalized recommendation-based MiRNA-Disease Association prediction (PRMDA) to predict potential miRNA-disease associations. The core idea for PRMDA is to construct a new rating matrixes by implementing personalized recommendation-based algorithm on the integrated miRNA similarity matrix and integrated disease similarity matrix. The flowchart of PRMDA was shown in Figure 2. The source code of PRMDA could be downloaded from http://www.escience.cn/system/file?fileId=89408.

First of all, we built a new rating matrix for known miRNA-disease association network, which measures the importance of miRNA to disease and disease to miRNA for each miRNA-disease pair. The new rating matrix was derived from two submatrices for diseases and miRNA, respectively, we illustrated how one submatrix for diseases, denoted by *Z*_*d*_, was built. In order to clearly demonstrate the process of constructing *Z*_*d*_, we took the calculation of entity *Z*_*d*_ *(i, j)* for example, which represents the new association score between disease *d(i)* and miRNA *m(j)*. Before building new rating matrixes, we calculated two matrixes: integrated miRNA similarity matrix, denoted by *S*_*r*_, and integrated disease similarity matrix, denoted by *S*_*d*_. Then, we got the related-miRNA set *RM*_*i*_ for *d(i)*, which means that *RM*_*i*_ contains all *d(i)*-related miRNAs *m(k)* that satisfies *A(i, k)* = 1, where *A* is the miRNA-disease association adjacency matrix. Then, we counted the number of related diseases *d(t)* for *d(i)*, satisfying *S*_*d*_ *(d(t), d(i))* > 0, and denoted the number by variable *n*_*a*_. Next, we defined the dimension variable *N*_*a*_ as the total number of miRNAs. In the next step, we calculated the variable *n*_*tal*_. *n*_*tal*_ representing the number of miRNAs *m(k)*, which also had relationship with *m(j)*, in *RM*_*i*_, in other words, *S*_*m*_ *(m(k), m(j))*>0. The less *n*_*tal*_ is, the more specific the *m(j)* is. The personalized weight matrix for disease, *W*_*d*_, was established, where *W*_*d*_ *(i, j)*, the personalized miRNA *m(j)* weight for disease *d(i)*, was calculated as follows:

$$W_{d}\left(i,j\right)=n_{tal}\times \log\left(\frac{N_{a}}{n_{a}}\right)$$ (14)

Then, for the new rating submatrix for disease: *Z*_*d*_, the entity *Z*_*d*_ *(i, j)* was defined as below according to personalized weight:

$$Z_{d}\left(i,j\right)=\frac{W_{d}\left(i,j\right)}{\sum_{a=1}^{N_{a}}W_{d}\left(i,a\right)}$$ (15)

Similarly, the new rating submatrix for miRNA, denoted by *Z*_*m*_, was constructed in the same way with *Na*representing the number of diseases. In summary, the new rating submatrices for miRNA and diseases quantified the importance of disease for miRNA and miRNA for disease regarding each miRNA-disease association pair, respectively, by taking miRNA-related miRNAs and disease-related diseases information into consideration. After the construction of submatrices for both miRNA and disease, we normalized *Z*_*m*_ and *Z*_*d*_ to get final integrated rating matrix *Z*. We defined *Z* as follows:

$$Z\left(i,j\right)=\frac{Z_{m}\left(i,j\right)}{\left\|Z_{m}\left(i\right)\right\|}+\frac{Z_{d}\left(i,j\right)}{\left\|Z_{d}\left(i\right)\right\|}$$ (16)

where *Z* was considered to be the new rating matrix for miRNA-disease association network, which took both personalized weight for diseases and personalized weight for miRNAs into consideration.

Furthermore, we got *K*_*m*_ and *K*_*d*_ by multiplying integrated similarity for miRNAs and integrated similarity for diseases with miRNA-disease association adjacency matrix respectively, based on the assumption that miRNAs with similar functions tend to be related with diseases with similar phenotypes And we calculated matrix *K*, as the addition of normalized *K*_*m*_ and *K*_*d*_, as follows:

$$K\left(i,j\right)=\frac{K_{m}\left(i,j\right)}{\left\|K_{m}\left(i\right)\right\|}+\frac{K_{d}\left(i,j\right)}{\left\|K_{d}\left(i\right)\right\|}$$ (17)

Finally, the prediction matrix *R* was defined as below:

$$R\left(i,j\right)=\frac{K\left(i,j\right)}{\left\|K\left(i\right)\right\|}+\frac{Z\left(i,j\right)}{\left\|Z\left(i\right)\right\|}$$ (18)

where *R (i, j)* represents the rating for disease *d(i)* and miRNA *m(j)* association calculated by PRMDA.

## SUPPLEMENTARY MATERIALS TABLES












