# Supplementary material for: PRMDA: personalized recommendation-based MiRNA-disease association prediction
Source: Oncotarget. 2017 Sep 18;8(49):85568–83. doi: 10.18632/oncotarget.20996 (PMC5689632; doi:10.18632/oncotarget.20996)
Supplement: Supplementary file 1 [file oncotarget-08-85568-s001.pdf]

PRMDA: personalized recommendation-based MiRNA-disease association prediction

SUPPLEMENTARY MATERIALS

Supplementary Table 1: We provided a comparison table, comparing input, output and limitation for every computational miRNA-disease association prediction model mentioned in the manuscript. See Supplementary_Table_1

Supplementary Table 2: We further applied PRMDA to prioritize all the candidate miRNA-disease pairs based on all the miRNA-disease associations recorded in HMDD database as training samples. Prediction results were publicly released for further research and experimental validation. See Supplementary_Table_2

Supplementary Table 3: We provided disease semantic similarity matrix, constructed according to disease semantic similarity model 1. See Supplementary_Table_3

Supplementary Table 4: We provided disease semantic similarity matrix, constructed according to disease semantic similarity model 2. See Supplementary_Table_4

Supplementary File 1: We provided a zip file, containing the rank of all miRNA-disease association pairs for each disease in separate file. See Supplementary_File_1
